# Safe needling depths of upper back acupoints in children: a retrospective study

**DOI:** 10.1186/s12906-016-1060-x

**Published:** 2016-02-27

**Authors:** Yi-Chun Ma, Ching-Tien Peng, Yu-Chuen Huang, Hung-Yi Lin, Jaung-Geng Lin

**Affiliations:** Graduate Institute of Chinese Medicine, College of Chinese Medicine, China Medical University, Taichung, Taiwan, ROC; Department of Pediatrics, Tai-An Hospital, Taichung, Taiwan; Children’s Hospital of China Medical University, Taichung, Taiwan, ROC; School of Chinese Medicine, College of Chinese Medicine, China Medical University, Taichung, Taiwan, ROC; Department of Medical Research, China Medical University Hospital, Taichung, Taiwan, ROC; China Medical University, No.91, Xueshi Rd., North Dist., Taichung City, 40402 Taiwan, ROC

**Keywords:** Acupuncture, Safe depth, Children, Upper back

## Abstract

**Background:**

Acupuncture is applied for treating numerous conditions in children, but few studies have examined the safe needling depth of acupoints in the pediatric population. In this study, we investigated the depths to which acupuncture needles can be inserted safely in the upper back acupoints of children and the variations in safe depth according to sex, age, weight, and body mass index (BMI).

**Methods:**

We retrospectively studied computed tomography (CT) images of patients aged 4 to 18 years who underwent chest CT at China Medical University Hospital between December 2004 and May 2013. The safe depths of 23 upper back acupoints in the Governor Vessel (GV), Bladder Meridian (BL), Small Intestine Meridian (SI), Gallbladder Meridian (GB) and Spleen Meridian (SP) were measured directly from the CT images. The relationships between the safe depths of these acupoints and sex, age, body weight, and BMI were analyzed.

**Results:**

The results indicated significant differences in safe needling depth between boys and girls in most upper back acupoints, except at BL42, BL44, BL45, BL46, GB21 and SP21. Safe depths differed significantly depending on age (*p* < 0.001), weight (*p* ≤ 0.01), and BMI (*p* < 0.05). Multiple regression analysis revealed that weight was the most crucial factor in determining the safe depth.

**Conclusions:**

Sex, age, weight, and BMI are relevant factors in determining the safe needling depths of upper back acupoints in children. Physicians should pay attention to wide variations in needle depth when performing acupuncture.

## Background

Acupuncture is a traditional Chinese medical practice that has been used to treat numerous diseases in China for thousands of years. The practice has gained popularity in Western countries and become one of the most popular forms of complementary and alternative therapy. Acupuncture has been applied to treat several conditions in children, including pain, nocturnal enuresis, postoperative nausea and vomiting, allergic rhinitis, laryngospasm, and neurological disorders [1, 2]. Thus, the safety of acupuncture is critical and should be considered carefully regarding pediatric patients.

Acupuncture may cause serious adverse events including subarachnoid hemorrhages, pneumothorax, cardiac ruptures, nerve impairment, intestinal obstruction, hemoptysis, reversible comas, and infection [3]. Pneumothorax is the most frequent organ injury caused by acupuncture [4]. Acupoints over upper back used to be applied for cough, dyspnea, backache and shoulder pain. Hence, studying the safe depths of acupoints over upper back is critical to prevent serious and common complications, such as pneumothorax from acupuncture.

The depths of acupunture needling recorded in ancient references have variations and there was no difference between different body sizes. Besides, no references were specific for children. In clinical practice, the depths of needling were usually performed according to practitioners’ experiences and patients’ responses. However, children usually cannot response properly to the needling stimulation. There were risks for serious complications such as pneumothorax or organ damage. Therefore, we want to establish data of the safe needling depth, considering the factors of sex, age, weight and BMI in children.

In children, the physical development process may cause changes in lean muscle mass and fat volume [5]. Particularly during puberty, the sexual dimorphism of body composition and the wide time range of pubertal onset cause wide variations in fat volume, muscle mass, and their distribution [6]. Such variations may be highly complex and may influence safe needling depth. Such difficulties are not encountered when treating adults.

Most previous studies on safe needling depth have involved small sample sizes and adult groups [7, 8]. One study measured the safe depths of back loci in adults, finding differences depending on body size but not sex [9]. Few studies have evaluated safe needling depth in children. Only two studies measuring the safe depths and therapeutic depths of abdomen acupoints have included pediatric populations [10, 11]. In this study, we included large sample sizes of children and measured the safe depths of upper back acupoints by analyzing computed tomography (CT) scans. We also evaluated the variations in safe depth according to sex, age, weight, and body mass index (BMI).

## Methods

### Study population

All patients aged 4 to 18 years who underwent chest CT between December 2004 and May 2013 at China Medical University Hospital (CMUH) were identified. These patients underwent CT scans for evaluating an acute chest or upper back condition such as acute accidental injuries, pneumothorax, pneumonia, and cardiac diseases. Patients with back trauma or chronic oncological diseases were excluded because of the possible effect on the thickness of subcutaneous tissues and muscles in the back. Thus, we included 4 to 18 years patients who underwent chest CT between December 2004 and May 2013 at CMUH without back trauma or chronic oncological diseases.

The age, sex, height and body weight of each patient were obtained from chart records. BMI was measured according to weight (kg)/height^2^ (m^2^). Patients were divided into five groups according to age in years: 4–6, 7–9, 10–12, 13–15, and 16–18 on the basis of previous references [10, 11]. Patients were also divided into four weight groups according to growth charts for Taiwanese children and adolescents [12]: below the third percentile, from the third percentile up to the 85th percentile, from the 85th percentile up to the 97th percentile, and at or above the 97th percentile. Patients were divided into four BMI groups according to the same charts: underweight, defined as below the fifth percentile; healthy weight, defined as from the fifth percentile up to the 85th percentile; overweight, defined as from the 85th percentile up to the 95th percentile; and obese, defined as at or above the 95th percentile [12, 13]. The data were anonymized and this study was approved by the Research Ethics Committee of CMUH.

### Measurement of safe depths at upper back acupoints

Acupoints were located according to a classic Chinese acupuncture technique called Tong Shen Cun (cun). The back transverse Tong Shen Cun is one-sixth of the shortest distance between the two scapulae. The back vertical Tong Shen Cun is located using vertebral spinous processes. The safe depths of acupoints in the Governor Vessel (GV) are defined as the distance from the skin surface of the acupoint to the epidural layer. The safe depths of other acupoints in the upper back are defined as the distance from the skin surface of acupoints to the pleura.

**Fig. 1:**
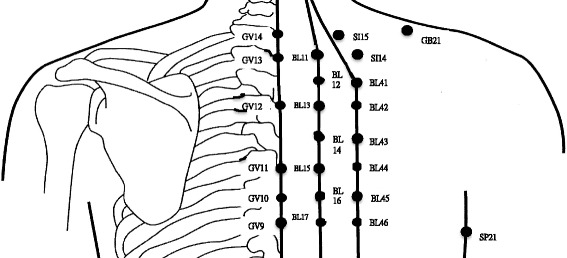
Total 23 acupoints over upper back. The figure was adapted from “Review on the history and practice of the needling depth of acupoints” [25]

Twenty-three back acupoints located in the GV, Bladder Meridian (BL), Small Intestine Meridian (SI), Gallbladder Meridian (GB) and Spleen Meridian (SP) were measured. Patients diagnosed with pneumothorax were measured on the healthy side of their backs. GV14, GV13, GV12, GV11, GV10, and GV9 were located at the central points between the spinous processes of C7 and T1, T1 and T2, T3 and T4, T5 and T6, T6 and T7, and T7 and T8, respectively. The BL was 1.5 and 3 cun lateral to the GV. For example, BL13 and BL42 were 1.5 and 3 cun lateral to the GV12. SI15 was 2 cun lateral to GV14 and SI14 was 3 cun lateral to GV13. GB21 was located at the midpoint of the line between the C7 and the lateral end of acromion. Sp21 was located at intersection of the sixth intercostal space and the midaxillary line (Fig. 1).

The CT machines used at CMUH were the Optima Speed CT Scanner (GE Healthcare, General Electric, USA), Optima CT660 (GE Healthcare) and Light Speed 16 CT Scanner (GE Healthcare). All CT images were captured in the transverse plane and body positions of all the participants were supine. The section thickness between each image was 5 mm. Safe depths were measured by examining the CT images on a Picture Archiving and Communication System (PACS) monitor (Realsync, Taiwan).

### Statistical analysis

The safe depths of acupoints among different age, weight, and BMI groups were analyzed using a one-way analysis of variance. Student’s t tests were used to compare the safe depths of acupoints between boys and girls. Multiple regression models were used to analyze whether sex, age, weight, or BMI are relevant factors in determining the safe depths of acupoints. Statistical analyses were performed using the SPSS software package, Version 18.0 (SPSS Inc., Chicago, IL), and *p* < 0.05 was considered significant.

## Results

A total of 319 patients (205 boys and 114 girls) aged 4 to 18 years were included in this study. The general characteristics of the patients are shown in Table 1.

**Table 1:** General characteristics of patients

Characteristics	Subjects numbers
Gender	
Male	205
Female	114
Age (years old)	
4–6	79
7–9	45
10–12	30
13–15	56
16–18	109
Weight	
<3rd percentile	32
3–<85th percentile	215
85–<97th percentile	42
≥97th percentile	29
BMI	
Underweight	52
Healthy weight	186
Overweight	18
Obesity	30

The mean and standard deviation of the safe depths of boys and girls at 23 back acupoints are listed in Table 2. The shallowest and greatest depths of both sexes were at BL46 and SI15, respectively. Among the upper back acupoints, the deepest safe depth was 2.13 times in boys and 2.05 times in girls than the shallowest one. The safe depths of most back acupoints differed significantly between boys and girls, except BL42, BL44, BL45, BL46, GB21 and SP21. All 23 acupoints in boys were deeper than in girls.

**Table 2:** Safe depths of 23 upper back acupoints of sexes

Acupoints	Boys	Girls	*P* value
	Mean ± S.D.	Mean ± S.D.	
GV9	27.78 ± 8.88	24.87 ± 7.83	0.003^a^
GV10	28.54 ± 8.47	26.02 ± 7.67	0.009^a^
GV11	28.17 ± 8.21	25.88 ± 7.04	0.009^a^
GV12	30.45 ± 9.90	28.03 ± 8.24	0.028^a^
GV13	33.61 ± 10.07	30.59 ± 9.11	0.008^a^
GV14	33.81 ± 10.57	30.70 ± 10.68	0.012^a^
BL11	33.39 ± 10.48	28.95 ± 8.67	<0.001^a^
BL12	35.61 ± 9.61	32.33 ± 8.09	0.001^a^
BL13	28.54 ± 7.08	26.41 ± 5.38	0.003^a^
BL14	24.92 ± 7.95	22.69 ± 6.63	0.008^a^
BL15	25.48 ± 6.57	23.93 ± 5.41	0.024^a^
BL16	24.15 ± 7.19	21.89 ± 6.24	0.004^a^
BL17	25.37 ± 6.87	23.62 ± 6.20	0.021^a^
BL41	30.86 ± 9.20	28.21 ± 7.89	0. 010^a^
BL42	28.02 ± 8.69	26.48 ± 7.25	0.110
BL43	24.68 ± 7.54	22.95 ± 6.75	0.043^a^
BL44	20.31 ± 7.79	19.19 ± 7.01	0.202
BL45	18.45 ± 7.07	17.62 ± 6.50	0.300
BL46	18.16 ± 6.63	16.96 ± 6.12	0.111
SI14	36.82 ± 12.23	32.51 ± 10.81	0.002^a^
SI15	38.75 ± 12.10	34.74 ± 10.47	0.003^a^
GB21	28.01 ± 9.37	26.61 ± 8.81	0.192
SP21	19.48 ± 4.39	18.84 ± 4.56	0.216

Unit of mean ± SD: mm

^a^Statistically significant

Safe depths significantly differed among age groups (*p* < 0.001; Table 3). Almost all safe depths significantly correlated with an increase in age. The safe depth was greater in those who are older.

**Table 3:** Safe depths of 23 upper back acupoints of age groups

Points	4–6 y/o (*n* = 79)	7–9 y/o (*n* = 45)	10–12 y/o (*n* = 30)	13–15 y/o (*n* = 56)	16–18 y/o (*n* = 109)	*P* value
	Mean ± S.D.	Mean ± S.D.	Mean ± S.D.	Mean ± S.D.	Mean ± S.D.	
GV9	19.31 ± 2.84	23.32 ± 6.17	27.20 ± 7.54	29.83 ± 8.04	31.83 ± 8.55	<0.001^a^
GV10	20.70 ± 3.62	23.64 ± 4.93	28.86 ± 6.12	29.88 ± 7.74	32.84 ± 8.31	<0.001^a^
GV11	22.14 ± 4.80	22.96 ± 4.52	27.70 ± 6.19	29.56 ± 7.56	31.71 ± 8.29	<0.001^a^
GV12	22.26 ± 4.54	25.93 ± 5.59	30.75 ± 7.58	32.40 ± 8.73	34.63 ± 10.16	<0.001^a^
GV13	23.02 ± 5.59	29.18 ± 7.70	35.75 ± 8.25	35.78 ± 7.99	38.26 ± 8.65	<0.001^a^
GV14	23.26 ± 4.95	26.57 ± 8.05	33.20 ± 9.48	37.83 ± 8.79	39.29 ± 9.54	<0.001^a^
BL11	24.93 ± 5.44	28.71 ± 8.00	31.29 ± 7.86	36.07 ± 10.53	36.01 ± 10.63	<0.001^a^
BL12	28.54 ± 8.50	32.57 ± 8.48	34.43 ± 8.24	37.74 ± 8.56	37.78 ± 8.33	<0.001^a^
BL13	24.82 ± 2.74	25.01 ± 3.00	26.53 ± 4.55	29.17 ± 7.57	30.68 ± 8.04	<0.001^a^
BL14	18.90 ± 2.66	21.35 ± 4.32	24.67 ± 6.53	26.42 ± 8.14	27.71 ± 8.48	<0.001^a^
BL15	20.94 ± 3.46	23.42 ± 4.57	24.45 ± 5.36	26.72 ± 6.45	27.65 ± 6.78	<0.001^a^
BL16	18.23 ± 2.76	21.12 ± 5.04	23.96 ± 5.37	25.32 ± 7.26	26.79 ± 7.50	<0.001^a^
BL17	19.14 ± 2.85	22.56 ± 4.03	25.60 ± 5.33	26.98 ± 6.76	28.33 ± 6.89	<0.001^a^
BL41	24.77 ± 3.51	25.32 ± 5.99	27.74 ± 6.44	33.75 ± 8.96	34.17 ± 9.95	<0.001^a^
BL42	21.97 ± 3.88	24.86 ± 5.50	28.20 ± 7.28	29.49 ± 8.15	31.30 ± 9.28	<0.001^a^
BL43	19.41 ± 3.74	21.92 ± 4.32	24.29 ± 6.19	25.60 ± 7.95	27.46 ± 8.14	<0.001^a^
BL44	15.21 ± 5.67	18.72 ± 5.42	19.68 ± 5.99	21.65 ± 7.61	22.99 ± 8.04	<0.001^a^
BL45	13.14 ± 3.39	15.84 ± 4.47	19.43 ± 6.47	20.26 ± 6.46	21.32 ± 7.53	<0.001^a^
BL46	12.74 ± 2.35	15.24 ± 3.58	19.34 ± 6.04	19.53 ± 6.45	21.01 ± 7.00	<0.001^a^
SI14	26.30 ± 6.29	30.88 ± 9.25	34.18 ± 10.28	40.32 ± 11.41	41.31 ± 11.91	<0.001^a^
SI15	26.92 ± 4.94	31.77 ± 7.65	39.52 ± 9.72	43.85 ± 12.10	43.17 ± 10.54	<0.001^a^
GB21	20.84 ± 3.25	23.70 ± 4.99	30.16 ± 9.14	28.94 ± 8.97	32.45 ± 10.06	<0.001^a^
SP21	16.97 ± 2.89	16.88 ± 2.18	19.89 ± 3.25	19.99 ± 4.44	21.34 ± 5.17	<0.001^a^

Unit of mean ± SD: mm

^a^Statistically significant

Safe depths significantly differed between weight groups (*p* < 0.01; Table 4) and significantly increased with weight at all 23 acupoints. Safe depths among those weighing above the 97th percentile were between 1.19 (BL12) and 1.65 (BL45) times deeper than among those weighing below the third percentile.

**Table 4:** Safe depths of 23 upper back acupoints in different weight groups

Points	<3rd (*n* = 32)	3–<85th (*n* = 215)	85–<97th (*n* = 42)	≥97th (*n* = 29)	*P* value
	Mean ± S.D.	Mean ± S.D.	Mean ± S.D.	Mean ± S.D.	
GV9	23.95 ± 5.74	26.00 ± 7.55	29.07 ± 9.61	32.00 ± 13.69	<0.001^a^
GV10	25.88 ± 5.85	26.45 ± 7.16	30.51 ± 8.18	34.18 ± 13.32	<0.001^a^
GV11	24.98 ± 5.10	26.41 ± 6.55	29.28 ± 8.97	34.25 ± 12.74	<0.001^a^
GV12	27.58 ± 6.53	28.12 ± 6.65	32.47 ± 9.81	38.48 ± 19.01	<0.001^a^
GV13	30.21 ± 8.23	31.25 ± 8.44	35.47 ± 9.16	40.42 ± 16.13	<0.001^a^
GV14	31.31 ± 9.68	31.66 ± 9.36	34.88 ± 12.04	38.63 ± 16.11	0.004^a^
BL11	29.99 ± 9.18	30.87 ± 8.57	33.96 ± 10.81	37.51 ± 16.68	0.003^a^
BL12	33.08 ± 9.92	33.58 ± 8.33	36.21 ± 8.89	39.23 ± 12.94	0.007^a^
BL13	26.25 ± 4.70	26.96 ± 4.99	29.26 ± 6.82	33.51 ± 12.84	<0.001^a^
BL14	21.86 ± 5.44	23.45 ± 6.48	25.99 ± 8.42	28.99 ± 12.37	<0.001^a^
BL15	22.86 ± 4.42	24.13 ± 5.20	27.28 ± 6.72	29.73 ± 10.12	<0.001^a^
BL16	21.36 ± 5.25	22.50 ± 5.90	25.42 ± 7.59	28.89 ± 10.86	<0.001^a^
BL17	22.23 ± 4.57	24.13 ± 6.07	26.70 ± 6.78	29.35 ± 9.86	<0.001^a^
BL41	26.45 ± 5.32	29.14 ± 7.03	31.83 ± 10.67	36.77 ± 15.45	<0.001^a^
BL42	24.59 ± 5.71	26.38 ± 6.55	29.53 ± 6.86	35.74 ± 15.50	<0.001^a^
BL43	22.17 ± 4.95	23.00 ± 5.34	26.11 ± 8.25	30.98 ± 13.86	<0.001^a^
BL44	17.10 ± 4.24	19.06 ± 6.13	22.28 ± 8.10	25.90 ± 13.45	<0.001^a^
BL45	15.01 ± 4.16	17.28 ± 5.57	20.52 ± 7.03	24.72 ± 11.74	<0.001^a^
BL46	15.41 ± 4.58	16.80 ± 4.87	20.12 ± 6.69	23.66 ± 12.14	<0.001^a^
SI14	31.64 ± 9.57	33.98 ± 9.68	37.57 ± 12.13	45.52 ± 20.78	<0.001^a^
SI15	35.12 ± 10.03	36.39 ± 10.09	38.22 ± 12.86	45.15 ± 18.52	0.001^a^
GB21	25.40 ± 9.45	26.32 ± 7.88	31.09 ± 9.68	33.27 ± 13.27	<0.001^a^
SP21	17.97 ± 4.26	18.83 ± 4.12	20.26 ± 4.15	22.46 ± 5.89	<0.001^a^

Unit of mean ± SD: mm

^a^Statistically significant

Safe depths also significantly varied between BMI groups (*p* < 0.05; Table 5). An increase in BMI was significantly correlated with increased safe depth. Safe depths in the obese group were between 1.18 (BL12) and 1.47 (BL44) times deeper than in the underweight group.

**Table 5:** Safe depth of 23 upper back acupoints of BMI groups

Points	Underweight^a^ (*n* = 52)	Healthy weight^b^ (*n* = 186)	Overweight^c^ (*n* = 18)	Obesity^d^ (*n* = 30)	*P* value
	Mean ± S.D.	Mean ± S.D.	Mean ± S.D.	Mean ± S.D.	
GV9	24.40 ± 5.94	26.21 ± 7.73	29.42 ± 10.63	30.92 ± 12.99	0.003^e^
GV10	25.27 ± 5.47	26.75 ± 7.21	30.51 ± 8.62	33.58 ± 12.14	<0.001^e^
GV11	25.23 ± 5.02	26.38 ± 6.94	30.47 ± 8.26	33.40 ± 11.82	<0.001^e^
GV12	26.91 ± 5.80	28.54 ± 6.89	32.23 ± 9.25	36.78 ± 17.38	<0.001^e^
GV13	30.62 ± 7.26	31.61 ± 8.36	34.86 ± 9.39	38.70 ± 14.71	<0.001^e^
GV14	30.26 ± 9.19	31.84 ± 9.39	33.86 ± 13.01	38.80 ± 14.75	0.002^e^
BL11	30.25 ± 8.39	30.90 ± 8.96	33.94 ± 11.20	37.66 ± 15.18	0.003^e^
BL12	32.78 ± 9.13	33.86 ± 8.52	33.92 ± 8.54	38.57 ± 11.70	0.037^e^
BL13	26.09 ± 4.78	27.06 ± 4.89	28.94 ± 7.44	33.22 ± 12.10	<0.001^e^
BL14	22.72 ± 5.91	23.33 ± 6.60	26.68 ± 8.13	29.00 ± 11.85	<0.001^e^
BL15	22.62 ± 3.81	24.57 ± 5.35	26.70 ± 6.67	29.78 ± 9.61	<0.001^e^
BL16	21.98 ± 5.31	22.37 ± 6.17	26.30 ± 6.86	28.62 ± 10.07	<0.001^e^
BL17	23.21 ± 4.98	24.20 ± 6.00	26.35 ± 6.94	29.03 ± 9.72	<0.001^e^
BL41	27.33 ± 5.67	29.16 ± 7.41	32.41 ± 10.18	35.88 ± 14.86	<0.001^e^
BL42	25.42 ± 6.23	26.60 ± 6.51	30.06 ± 6.90	32.92 ± 13.70	<0.001^e^
BL43	21.98 ± 4.87	22.90 ± 5.56	27.11 ± 6.64	30.25 ± 13.17	<0.001^e^
BL44	17.59 ± 4.86	18.99 ± 6.29	23.29 ± 7.90	25.85 ± 12.07	<0.001^e^
BL45	15.98 ± 4.77	17.42 ± 5.63	21.73 ± 6.97	22.89 ± 11.49	<0.001^e^
BL46	16.15 ± 4.54	16.94 ± 5.00	19.67 ± 6.69	22.71 ± 11.53	<0.001^e^
SI14	32.40 ± 9.29	33.88 ± 9.94	39.87 ± 12.20	42.61 ± 18.58	<0.001^e^
SI15	35.25 ± 9.01	36.41 ± 10.49	39.54 ± 11.60	45.26 ± 18.02	<0.001^e^
GB21	26.43 ± 8.52	26.67 ± 8.11	31.23 ± 11.01	31.41 ± 12.84	0.012^e^
SP21	19.36 ± 4.86	18.76 ± 4.03	20.40 ± 4.55	20.68 ± 5.50	0.086

Unit of mean ± SD: mm

^a^Underweight: patients with BMI below the fifth percentile

^b^Healthy weight: patients with BMI from the 5th percentile up to the 85th percentile

^c^Overweight: patients with BMI from the 85th percentile up to the 95th percentile

^d^Obese: patients with BMI at or above the 95th percentile

^e^Statistically significant

We performed multiple regression models to analyze whether age, sex, weight, and BMI are relevant factors in determining the safe depths of acupoints. The results revealed that weight was significantly correlated with safe depth at all 23 acupoints. (Table 6) Thus, weight was the most crucial factor in determining the safe depths of upper back acupoints.

**Table 6:** Multiple regression analysis for safe depths of 23 upper back acupoints

Points	Variables in multiple regression model			
	Age	Weight	BMI	Sex (male vs female)
	β (95 % CI)	β (95 % CI)	β (95 % CI)	β (95 % CI)
	*P* value	*P* value	*P* value	*P* value
GV9	−0.06 (−0.38–0.26) 0.716	0.33 (0.22–0.43) <0.001*	0.08 (−0.16–0.32) 0.527	−0.60 (−2.15–0.95) 0.446
GV10	−0.01 (−0.31–0.28) 0.927	0.28 (0.18–0.38) <0.001*	0.23 (0.01–0.45) 0.038*	−0.85 (−2.27–0.57) 0.241
GV11	−0.25 (−0.55–0.05) 0.100	0.31 (0.22–0.41) <0.001*	0.19 (−0.04–0.41) 0.098	−1.05 (−2.49–0.39) 0.152
GV12	−0.14 (−0.48–0.20) 0.412	0.34 (0.23–0.45) <0.001*	0.21 (−0.04–0.47) 0.101	−1.41 (−3.06–0.24) 0.093
GV13	0.27 (−0.07–0.60) 0.114	0.27 (0.16–0.38) <0.001*	0.27 (0.02–0.52) 0.035*	−0.82 (−2.45–0.81) 0.324
GV14	0.46 (0.05–0.86) 0.027*	0.26 (0.13–0.39) <0.001*	0.17 (−0.13–0.48) 0.261	−0.61 (−2.58–1.36) 0.544
BL11	−0.07 (−0.50–0.37) 0.757	0.29 (0.15–0.44) <0.001*	0.10 (−0.22–0.43) 0.531	1.34 (−0.78–3.46) 0.213
BL12	0.18 (−0.24–0.60) 0.395	0.19 (0.06–0.33) 0.005*	0.02(−0.30–0.33) 0.927	1.06 (−0.97–3.09) 0.304
BL13	−0.51 (−0.78–-0.24) <0.001*	0.31 (0.22–0.40) <0.001*	0.04 (−0.16–0.24) 0.700	−0.52 (−1.83–0.80) 0.442
BL14	−0.32 (−0.62– −0.01) 0.044*	0.32 (0.22–0.42) <0.001*	0.037 (−0.19–0.27) 0.755	−0.82 (−2.31–0.68) 0.284
BL15	−0.33 (−0.58– −0.09) 0.008*	0.25 (0.17–0.33) <0.001*	0.20 (0.01–0.38) 0.035*	−0.77 (−1.96–0.42) 0.201
BL16	−0.16 (−0.44–0.12) 0.253	0.24 (0.15–0.33) <0.001*	0.20 (−0.01–0.40) 0.065	−0.142 (−1.48–1.20) 0.835
BL17	−0.22 (−0.47–0.03) 0.078	0.29 (0.21–0.37) <0.001*	0.05 (−0.14–0.23) 0.609	−1.16 (−2.36–0.04) 0.057
BL41	−0.51 (−0.86– −0.16) 0.005*	0.43 (0.31–0.54) <0.001*	−0.08 (−0.35–0.19) 0.553	−1.14 (−2.86–0.57) 0.191
BL42	−0.32 (−0.64– −0.01) 0.046*	0.33 (0.23–0.44) <0.001*	0.06 (−0.17–0.30) 0.598	−1.69 (−3.23– −0.14) 0.032*
BL43	−0.32 (−0.62– −0.02) 0.036*	0.28 (0.19–0.38) <0.001*	0.13 (−0.09–0.36) 0.242	−0.97 (−2.42–0.47) 0.186
BL44	−0.36 (−0.68– −0.04) 0.026*	0.29 (0.18–0.39) <0.001*	0.138 (−0.10–0.38) 0.256	−1.62 (−3.17– −0.07) 0.040*
BL45	−0.40 (−0.67– −0.14) 0.003*	0.34 (0.25–0.42) <0.001*	0.00 (−0.20–0.20) 1.000	−2.25 (−3.53– −0.98) 0.001*
BL46	−0.31 (−0.55– −0.07) 0.012*	0.31 (0.23–0.38) <0.001*	−0.01 (−0.19–0.18) 0.938	−1.66 (−2.84– −0.47) 0.006*
SI14	−0.10 (−0.56–0.36) 0.674	0.41 (0.26–0.56) <0.001*	0.13 (−0.22–0.47) 0.473	0.03 (−2.21–2.27) 0.978
SI15	0.20 (−0.26–0.66) 0.383	0.34 (0.20–0.49) <0.001*	0.18 (−0.16–0.53) 0.300	−0.22 (−2.45–2.00) 0.846
GB21	−0.26 (−0.64–0.13) 0.189	0.39 (0.26–0.51) <0.001*	−0.17 (−0.46–0.12) 0.249	−2.12 (−3.99– −0.25) 0.026*
SP21	−0.04 (−0.24–0.16) 0.695	0.14 (0.08–0.21) <0.001*	−0.07 (−0.22–0.09) 0.389	−0.57 (−1.55–0.41) 0.254

*Statistically significant β regression coefficient, *CI* confidence interval

## Discussion

Performing acupuncture on back acupoints has been demonstrated to be an effective alternative therapy for local myofascial pain [14, 15], asthma, allergic rhinitis [16], and tuberculosis consumptive diseases [17]. As the popularity of acupuncture increases, the importance of determining the safe depths of acupoints also increases, particularly the safe depths in the upper back. Acupuncture on the upper back may cause pleural and lung injuries [18, 19]. In pediatric groups, safe needling depths vary largely because of rapid changes in body size and shape. This study is the first to estimate the variations in the safe depths of upper back acupoints according to sex, age, weight, and BMI in children aged 4 to 18 years. We observed boys had deeper safe depth than girls. The safe depths were greater among older groups, heavier weight groups, and higher BMI groups.

The safe depths of all upper back acupoints in boys were deeper than in girls, and all such differences were significant, except at BL42, BL44, BL45, BL46, GB21 and SP21. Girls tend to accumulate more total body and subcutaneous fat, which is deposited mainly in the gynoid region and thigh during and after puberty. However, among boys, more fat is deposited in the upper segment of the body, both subcutaneously and intraabdominally, and more total lean and muscle mass is developed in this period [5, 20]. The greater amount of muscle mass and trunk fat among boys could explain this difference between the sexes, which has not been observed in studies on pediatric abdomen acupoints [10] or adult back acupoints [9].

Safe depth significantly differed among age groups and was significantly correlated with an increase in age for nearly all acupoints. Age influences safe depths because muscle mass and subcutaneous adipose tissue increase with age [5] among children. However, age need not be considered when determining the safe depths in adults.

Safe depths significantly differed between weight groups and significantly increased with weight at all 23 acupoints. Thicker fat or muscle tissue layers in heavier children may explain this observation, which is compatible with the results of an adult study in which the safe depths of back acupoints were related to body size [9]. At BL42, BL44, BL45, and BL46, the safe depths of patients weighing above the 97th percentile were approximately 1.5–1.6 times the safe depths of those weighing below the third percentile. Clinicians should be aware of these weight-related differences and the risk of pneumothorax when performing acupuncture in this area of the body.

The BMI characterizes the relative proportion of child’s weight and height percentile for age and sex. The BMI is the optimal clinical standard for diagnosing obesity in children and adolescents [21, 22]. We found that increasing BMI was significantly correlated with increased safe depths. At BL43, BL44, BL45, and BL46, the safe depths in the obesity group were approximately 1.4–1.5 times deeper than those in the underweight group. This result was similar to that in the weight group.

Multiple regression analysis revealed that weight was the most critical factor in determining the safe depths of upper back acupoints. Previous studies have observed that BMI percentile changes may not accurately reflect changes in adiposity in children, particularly among male adolescents and children with lower BMIs [23]. The BMI is limited in its usefulness in predicting adiposity by its inability to distinguish fat mass from lean body mass in pediatric populations. We used growth charts for Taiwanese children and adolescents [12, 13] for decreasing ethnic differences, but BMI was also influenced by age, sex and pubertal status [24]. This might explain why BMI was less crucial than weight in influencing safe depths in this study.

This study has three noteworthy strengths. First, the study included larger pediatric sample sizes. Second, we studied factors such as sex, age, weight, and BMI that influence safe depth. Finally, the study determined safe depths by examining in vivo CT images, a more accurate method than recording measurements from cadavers. Depths from cadavers are unreliable because the tissues dries and contracts after freezing, anticorrosive positioning, and dyeing processes.

This study has limitations. First, the study used a retrospective design. Second, the examined children were patients, not healthy children. However, to increase validity, patients with diseases that might have affected the thickness of the subcutaneous tissues or muscles in their backs were excluded. Third, the sample size of the overweight group was small. Finally, this study was conducted in a single medical center, limiting its population generalizability.

## Conclusions

This study determined that the safe depths of most upper back acupoints significantly differ between the sexes. Safe depths significantly differed among age groups and significantly increased with weight and BMI. Acupuncturists should consider wide variations in safe needling depth in the upper backs of children to balance treatment effects and complications.

## Abbreviations

BMI: body mass index
CT: computed tomography
CMUH: China Medical University Hospital
GV: Governor Vessel
BL: bladder meridian
SI: small intestine meridian
GB: gallbladder meridian
SP: spleen meridian
